# Epigenetic Modulation of Estrogen Receptor Signaling in Ovarian Cancer

**DOI:** 10.3390/ijms26010166

**Published:** 2024-12-28

**Authors:** Maciej Skrzypczak, Ewa Wolinska, Łukasz Adaszek, Olaf Ortmann, Oliver Treeck

**Affiliations:** 1 Chair and Department of Gynecology, Medical University of Lublin, 20-954 Lublin, Poland; skrzypm@yahoo.co.uk; 2 Department of Pathology, Medical University of Warsaw, 02-091 Warsaw, Poland; ewa.wolinska7@gmail.com; 3 Clinic of Infectious Diseases, University of Life Sciences Lublin, 20-950 Lublin, Poland; lukasz.adaszek@up.lublin.pl; 4 Department of Gynecology and Obstetrics, University Medical Center Regensburg, 93935 Regensburg, Germany; olaf.ortmann@klinik.uni-regensburg.de

**Keywords:** epigenetics, ovarian cancer, methylation, histone modification, estrogen signaling, *ESR1*, *ESR2*, *GPER1*

## Abstract

Ovarian cancer remains one of the leading causes of cancer-related deaths in women. There are several processes that are described to have a causal relationship in ovarian cancer development, progression, and metastasis formation, that occur both at the genetic and epigenetic level. One of the mechanisms involved in its pathogenesis and progression is estrogen signaling. Estrogen receptors (ER) α, ERβ, and G-protein coupled estrogen receptor 1 (GPER1), in concert with various coregulators and pioneer transcription factors, mediate the effects of estrogens primarily by the transcriptional regulation of estrogen responsive genes, thereby exerting pleiotropic effects including the regulation of cellular proliferation and apoptosis. The expression and activity of estrogen receptors and their coregulators have been demonstrated to be regulated by epigenetic mechanisms like histone modifications and DNA methylation. Here, we intend to summarize and to provide an update on the current understanding of epigenetic mechanisms regulating estrogen signaling and their role in ovarian cancer. For this purpose, we reviewed publications on this topic listed in the PubMed database. Finally, we assess to which extent drugs acting on the epigenetic level might be suitable for the treatment of ovarian cancer.

## 1. Introduction

Ovarian cancer (OC) represents the fourth leading cause of cancer-related deaths in women worldwide, being responsible for 4.4% of cancer-related deaths in the world [1,2]. OC is a heterogeneous group of diseases categorized primarily into type I and type II tumors, each with distinct characteristics, prevalence, and prognostic implications [3]. Type I tumors include low-grade serous, endometrioid, clear cell, mucinous carcinomas, and malignant Brenner tumors. These tumors are typically slow growing, localized, and low-grade. However, clear cell carcinomas, though part of this group, are considered high-grade due to their aggressive behaviour [3]. Type I ovarian cancers are frequently associated with endometriosis and are less likely to involve *TP53* mutations. Common genetic alterations include mutations in *KRAS*, *BRAF*, *PTEN*, *PIK3CA*, and *ARID1A* [4]. Prognosis is generally favourable when detected early, as these tumors often progress slowly. Histological subtypes such as endometrioid, clear cell, and mucinous carcinomas are less frequent than type II tumors but exhibit regional variation; for instance, clear cell carcinoma is more common in East Asian populations. This type of OC is typically detected at early stages and has better outcomes due to its indolent nature. Type II tumors comprise high-grade serous carcinoma (HGSC), carcinosarcoma, and undifferentiated carcinoma. These tumors represent the majority of OC cases and are highly aggressive, with most originating from fallopian tube epithelial cells [5]. Type II OCs are characterized by widespread *TP53* mutations and genomic instability. Defects in homologous recombination repair, such as *BRCA1/2* mutations, are also prevalent. These tumors are frequently diagnosed at advanced stages, with rapid progression and poor prognosis despite aggressive treatment. HGSC accounts for approximately 70% of epithelial ovarian cancers, making it the most common and lethal subtype. The advanced presentation and aggressive behavior of type II tumors contribute to their poor prognosis, accounting for around 90% of ovarian cancer-related deaths [3]. The high mortality rate is caused by a variety of factors, such as the lack of symptoms that leads to late diagnosis, the lack of available screening tests, the development of drug resistance, and cancer recurrence [6,7]. The five-year survival rate largely depends on the tumor stage, reaching 89% at stage I, and rapidly decreasing to 20% at stage IV. Unfortunately, most OC patients are diagnosed at the later stages of tumor development [8]. The standard of care includes cytoreductive therapy and platinum-based chemotherapy, as well as adjuvant intraperitoneal therapy [9]. Endocrine therapy, using tamoxifen or aromatase inhibitors, which has become a standard treatment for estrogen receptor α (ERα)-positive breast cancer, has been shown to have limited efficacy in ERα-positive OC [10]. Currently, various efforts are underway to evaluate the effects of treatment regimens combining tamoxifen with novel anti-cancer drugs [11]. For example, it has been recently shown that the combination of tamoxifen and Gatipotuzumab has better outcomes than single drug treatment, and thus may provide a novel therapeutic strategy for OC [12]. After standard therapies, approximately 70% of OC patients have disease recurrence [13]. During the last twenty years, new technologies emerged that allowed a better understanding of the molecular changes that are related to OC development, progression, and therapy resistance [2]. These new techniques led to the identification of biomarkers with clinical use, i.e., *BRCA1/2* mutations, allowing the use of novel therapy regimens like PARP inhibitors such as Olaparib [2]. Another implication of genomic findings can be the optimization of therapeutic strategies. As was postulated by Gu et al., the primary debulking surgery followed by chemotherapy during the treatment of high-grade serous tubo-ovarian carcinoma can be more beneficial than chemotherapy treatment with surgery only after three to four cycles [14]. The authors of the cited work concluded that most patients carry chemo-resistant cancer cells at the time of diagnosis, and this strategy can better deplete these resistant cells. In addition to a growing number of molecular data that describe changes occurring throughout cancer development and progression at the level of classical gene regulation or DNA mutation, there are also growing multi-omics and mathematical models that gather this information and present a holistic analysis of the malfunction of cancer cells [15].

With the recognition of epigenetics’ crucial role in regulating physiological and pathological processes, the relevance of mechanisms like histone modification and DNA methylation in cancer, including OC, increasingly came into the focus of investigations. It is now commonly known that epigenetic factors can drive various cellular processes that can lead to the initiation, development, and progression of cancer as well as their chemoresistance to cancer drugs [16]. While treatment strategies targeting epigenetic alterations with specific drugs currently appear unfeasible, a deeper understanding of the role of epigenetic changes in OC development and progression and particularly their trigger mechanisms might lead to novel strategies for OC prevention.

## 2. Estrogen Signaling

**Figure 1:**
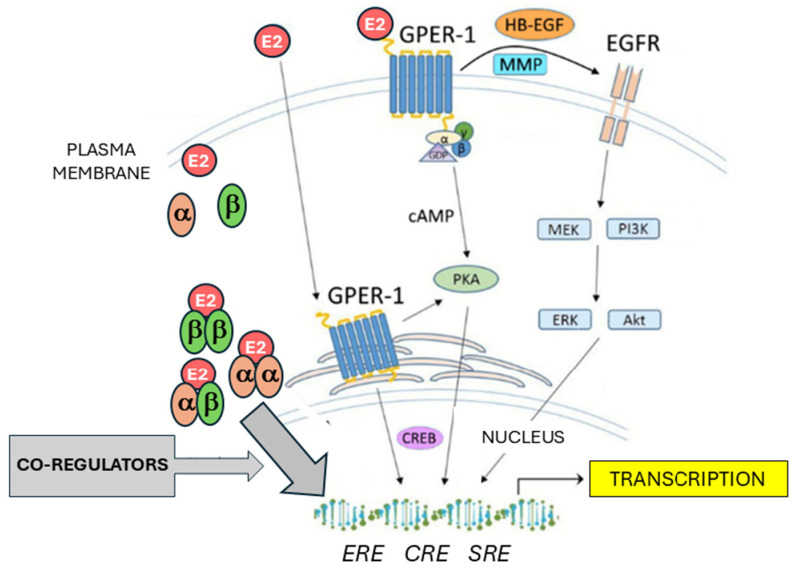
Schematic overview of estrogen signaling. E2: estradiol; α: ERα; β: ERβ; cAMP: cyclic adenosine monophosphate; PKA: protein kinase A; CREB: cAMP-response element binding protein; ERE: estrogen response element; CRE: cAMP-response element; SRE: serum response element; MMP: matrix metalloproteinase; HB-EGF: heparin-binding EGF-like growth factor; EGFR: epidermal growth factor receptor; MEK: mitogen-activated protein kinase; ERK: extracellular signal-regulated kinase; PI3K: phosphoinositide 3-kinase; Akt: protein kinase B (PKB). Further abbreviations are addressed in the text.

Estrogens represent one of the most important groups of sex steroid hormones in women. Among this group, the most widely distributed, and having active functions in multiple tissues, is 17β-estradiol (E2) [17]. Estrogen signaling is mediated through binding to estrogen receptors, among which the most important are the nuclear estrogen-activated transcription factors ERα and ERβ, coded by the genes *ESR1* and *ESR2*, respectively, and the transmembrane G protein-coupled estrogen receptor (*GPER1*) (Figure 1). E2 was found to have a high binding affinity for ERα, ERβ, and GPER1 [18]. Estrogens, upon binding to ERα or ERβ, trigger HSP90 dissociation and conformational changes enabling the formation of active ER homodimer or ERα/ERβ heterodimer complexes which enter the cell nucleus. Interacting with ER-coactivators or corepressors and pioneer factors like FOXA1 or GATA3, they act as a ligand-inducible transcription factor [19]. The main target DNA sequence for ER dimers is the estrogen response element (ERE). The resulting complex acts as a transcription factor that regulates gene transcription [20]. Estrogen signaling is dependent on the action of several coactivators and corepressors, as well as the ligand-independent activation of estrogen receptors [17]. These coregulators cannot bind to DNA directly, but they act via the interaction with DNA-bound estrogen receptor complexes. Coregulators can modify both histones and DNA, thus altering DNA accessibility to transcription factors. So far, more than 450 estrogen signaling coregulators have been discovered [21]. The best characterized transcriptional coactivators belong to the SRC/p160 family, consisting of SRC-1, SRC-2, and SCR-3 coactivators [22,23]. SRC proteins bound to estrogen receptor complexes can recruit further molecules—secondary coactivators [24]. Such secondary coactivators can be enzymes like histone methyltransferases or histone acetyltransferases [25]. The E2-driven regulation of gene transcription also depends on the interplay between coactivators and corepressors such as the NCoR1 (nuclear receptor corepressor 1)/SMRT (silencing mediator of retinoic acid and thyroid hormone receptor) corepressor family [21,26]. In addition to ERα and β, the G protein-coupled estrogen receptor (GPER1) mediates estrogen effects not through transcription factor binding to EREs, but via non-genomic signaling. This seven-transmembrane receptor, formerly known as GPR30, has several mechanisms of action. On the one hand, it mobilizes calcium and initiates cAMP synthesis. On the other hand, it transactivates the epidermal growth factor receptor (EGFR) which induces PI3K and MAPK signaling pathways and other mechanisms. GPER1 signaling ultimately leads to gene regulation affecting cell-cycle progression as well as proliferation, differentiation, apoptosis, migration, and invasion, making it an important player in carcinogenesis [27].

### Role of Estrogen Signaling in Ovarian Cancer

The ovaries are essential components of the female reproductive system, performing dual roles in gametogenesis and endocrine regulation. As endocrine glands, the ovaries synthesize and secrete steroid hormones, primarily estrogens and progesterone, which are critical for regulating the menstrual cycle, supporting pregnancy, and maintaining secondary sexual characteristics. Estrogen is produced predominantly by the granulosa cells of developing ovarian follicles. This hormone is vital for endometrial proliferation during the follicular phase of the menstrual cycle and contributes to the maintenance of bone density and cardiovascular health. The ovaries also produce small quantities of androgens, such as testosterone, which serve as precursors for estrogen synthesis through the aromatization process [28]. Estrogens do not only trigger physiological actions but have also been reported to play a role in the development and progression of OC [29]. Particularly in epithelial OC (EOC), estrogens have been shown to affect various cellular pathways including those involved in the regulation of proliferation, apoptosis, invasiveness, and epithelial-to-mesenchymal transition (EMT) [30]. The estrogen receptors, ERα, ERβ, and GPER1, are expressed both in normal ovarian tissue and in ovarian cancer [28]. At the mRNA level, the expression of the *ESR1* gene was detected in approximately 60% of OC tissues, whereas *ESR2* transcripts were the predominant ones in normal ovary tissue [31]. The *ESR1/ESR2* mRNA ratio was significantly increased in OC tissue. In a cell culture study employing primary cells, the *ESR1/ESR2* mRNA ratio in primary OC cells was ten times higher than in normal ovarian surface epithelium cultures, a finding that was basically corroborated on the protein level [32].

After initial controversies, mainly based on immunohistochemical (IHC) studies using unspecific antibodies, the tumor-suppressive role of the ERβ protein in OC became more and more obvious. In their IHC-based study, Lindgren et al. analyzed 53 benign, borderline, and malignant ovarian tumors of different types and found a significantly lower ERβ expression in OC tissue compared to normal ovaries [33]. Moreover, the level of ERβ protein expression in ovarian cancers has an impact on the survival of the patients. In the IHC-based study published by Halon et al., a higher ERβ expression (>30% of cells) was associated with increased overall survival time and progression-free time (*p* = 0.00161 and *p* = 0.03255, respectively) compared to patients with lower ERβ expression [34]. The role of ERα in OC has been considered controversial. In a large IHC-based study published by Sieh et al. in 2013, patients with OC of the endometrioid subtype survived significantly longer when their tumors expressed ERα [35]. However, in high-grade serous OC (HGSOC), no clear association of ERα expression with survival was reported, which is thought to result from the fact that HGSOC is typically driven by mutations in genes like *TP53* and has less reliance on hormone receptor signaling. Bogush et al. analyzed ER expression in serous OC by a quantitative immunofluorescence assay, and found that high expression levels of both ERα (≥25%) and ERβ (≥44%) predicted a significantly longer progression-free survival in patients after the first-line treatment of platinum and taxane-based adjuvant chemotherapy, which might primarily result from antitumoral ERβ action [36]. ERβ expression was found to be decreased not only in OC but also during the tumorigenesis of breast, colon, and prostate cancer [37,38,39]. In nude mice injected with ERβ-expressing ovarian BG1 cells, ERβ was able to strongly reduce the development of an orthotopic ovarian xenograft as well as the presence of tumor cells at the sites of metastasis, leading to an increased survival of the mice [40].

A multitude of in vitro studies employing OC cell lines revealed molecular mechanisms underlying the actions of ERα and ERβ in OC. The growth-promoting role of ERα, known from other cancer entities like breast cancer and mediated by the activation of proliferation genes, was also observed in OC. Furthermore, the activation of ERα was shown to trigger gene regulation patterns associated with the invasion and metastasis of OC cells [41]. Other in vitro studies elucidated the role of ERβ in this cancer entity. In studies including several from our group, ERβ was shown to exert tumor-suppressive effects like the decrease of growth and motility, but the activation of the apoptosis of OC cells, and transcriptome alterations, were identified underlying these actions [42,43]. Several highly specific ERβ agonists like ERB-041 and WAY200070 have been reported to trigger tumor suppressive responses in OC cells in vitro and could therefore be evaluated for efficacy in mouse models and clinical settings. In a recent study, another specific ERβ agonist was found to reduce the EMT and cancer stem cell (CSC) population in ovarian cancer. Given that ERα can trigger EMT and facilitates the maintenance of CSCs, the ERβ agonist was suggested to limit the CSC subpopulation with the potential to increase the survival of OC patients. In summary, regarding the two nuclear ERs, ERα is considered to mediate adverse E2 actions on OC cells, whereas ERβ is reported to predominantly exert tumor-suppressive actions in this cancer entity [44,45,46,47].

Studies on the role of the third ER, the non-nuclear, G protein-coupled estrogen receptor GPER1, in OC came to conflicting results, particularly those using IHC to detect this protein in OC tissues to correlate GPER1 protein levels with OC outcome. Several IHC-based studies suggested a tumor-promoting role of this receptor in OC [48,49]. In ovarian tissue samples, GPER1 was found to be broadly expressed in high-risk ovarian cancer, associated with lower 5-year survival rates [49]. In addition, its co-expression with EGFR was associated with shorter progression-free survival in OC patients [48]. However, there are also contrary data suggesting tumor-suppressive functions of GPER1 in ovarian malignancies. In a study by Ignatov et al., benign tumors and those of a low-malignant potential were found to have significantly higher GPER1 expression levels than the investigated OCs [50]. Early stage and well differentiated cancers strongly expressed GPER1, which was found in 83.1% of all malignant tumors. Moreover, they observed significantly longer disease-free survival for patients with GPER1-expressing OCs compared to those with GPER1-negative tumors [50]. In line with this, a study from our group reported that OC patients with tumors expressing high *GPER1* mRNA levels survived longer and had more lifetime without progression, when open-access mRNA and clinical data were analyzed [51]. A further study by Fraungruber et al. analyzing 156 OC samples supported a tumor suppressive role of GPER1. A high co-expression of *Dkk2* and *GPER1* was associated with better overall survival in OC patients [52]. These data suggest a prognostic relevance of both pathways, and indicate that therapeutic interventions targeting both estrogen and Wnt signaling pathways may be successful in OC. Taken together, current data suggest a tumor-promoting role of ERα, but anti-tumoral effects of ERβ and GPER1 in OC.

Estrogens are known to play an important role in the regulation of the immunological response [53]. In recent years, the importance of the interplay between tumor cells, stromal cells, immune cells, and extracellular molecules in the tumor microenvironment (TME) has been emphasized, as it has a profound effect on antitumor immunity and immunotherapeutic response. Both ERα and ERβ as well as aromatase, the key enzyme in the production of estrogens, are expressed in cells of the TME, like cancer-associated fibroblasts (CAFs) and tumor-associated macrophages (TAMs). In ovarian cancer, CAFs have been shown to overexpress ERα, which promotes tumor progression via paracrine signaling pathways [54]. The role of ERs in TAMs is an area of growing research interest, especially in the context of ovarian cancer. TAMs are highly plastic immune cells within the TME, and their behavior is modulated by various signals, including those mediated by estrogen and its receptors. In the ovarian tumor microenvironment, estrogen may contribute to the polarization of TAMs toward a pro-tumoral M2-like phenotype. This polarization supports tumor progression by promoting angiogenesis, immune suppression, and extracellular matrix remodeling [55,56].

## 3. Epigenetic Mechanisms

**Figure 2:**
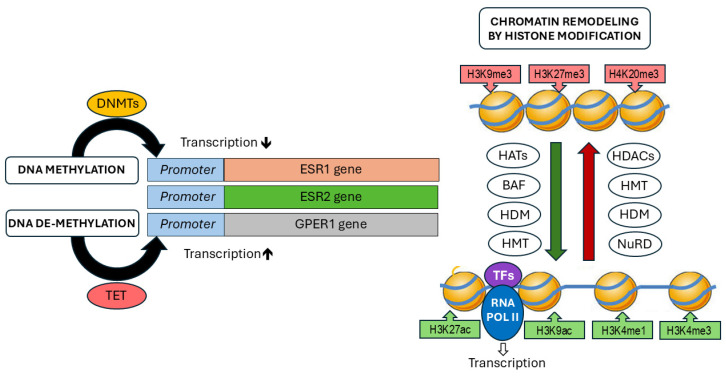
Schematic overview of DNA methylation and histone modifications modulating estrogen signaling. DNMTs: DNA methyltransferases, TET: Ten-Eleven Translocation Dioxygenases, HATs: histone acetyltransferases, BAF: BRG1/BRM-associated factor, HDM; histone demethylase, HMT: histone methyltransferase, HDACs: histone deacetylases, NuRD: Nucleosome Remodelling and Deacetylase Complex, TFs: transcription factors, RNA Pol II: RNA Polymerase II, H3K9me3: Histone H3 lysine 9 trimethylation, H3K27me3: Histone H3 lysine 27 trimethylation, H4K20me3: Histone H4 lysine 20 trimethylation, H3K27ac: Histone H3 lysine 27 acetylation, H3K9ac: Histone H3 lysine 9 acetylation, H3K4me3: Histone H3 lysine 4 trimethylation, H3K4me1: Histone H3 lysine 4 monomethylation.

Epigenetics refers to heritable changes in gene expression, which are primarily driven by mechanisms such as DNA methylation, histone modification, nucleosome positioning, and chromatin remodeling [57] (Figure 2). Some authors also consider non-coding RNAs (ncRNAs) as part of the epigenetic machinery, since epigenetic regulation is often described as a gene regulatory mechanism not altering the DNA sequence. However, there are compelling reasons not to classify ncRNAs as part of epigenetics. First, ncRNAs do not trigger heritable effects, but tend to function short-term, as observed with miRNAs and lncRNAs. Additionally, eukaryotic gene regulation, mediated by transcription factors or ncRNAs, does not involve changes to the DNA sequence. Therefore, ncRNAs can be considered as an additional mechanism of non-epigenetic gene regulation, primarily operating at the post-transcriptional level in eukaryotes [58,59].

DNA methylation is characterized by the addition of methyl groups to the fifth carbon of cytosine residues in CpG dinucleotides, typically within CpG islands. This methylation often occurs in the promoter regions of genes, where it plays a key role in regulating gene expression [60]. Several studies have reported differences in the methylation status between normal and tumor cells and tissues [61,62,63,64]. The methylation process is mediated by enzymes of the DNA methyltransferases group (DNMTs), and hypermethylation is usually related to transcriptional repression [65]. The opposite effect is induced by DNA demethylases, involving the Ten-Eleven Translocation Dioxygenases (TET), and leads to the removal of the methyl group from DNA sequences, leading to hypomethylation activating gene expression [66]. The second major mechanism in epigenetics is the posttranslational modification of histones [67]. Such changes usually occur at the histone end and can be caused by acetylation, methylation, ubiquitination, phosphorylation, SUMOylation, or ADP ribosylation. Histone modifications alter the configuration and density of chromatin, thereby influencing its accessibility to transcription factors and other components of the transcription machinery [68,69]. Among these modifications, those induced by histone methyltransferases (HMTs) and histone demethylases (HDMs) regulate histone methylation. Histone methylation can activate gene expression by the addition of epigenetic marks like H3K4me3 or suppress transcription by posting marks like H3K9me3. Another common epigenetic modification involves the addition or removal of acetyl groups at lysine residues in histones. This process is regulated by acetyltransferases (HATs) and histone deacetylases (HDACs), respectively. HATs are able to loosen chromatin structure and activate transcription by adding epigenetic marks like H4K16ac and H3K27ac. HDACs are classically associated with transcriptional repression by removing acetyl groups from histones, like H4K16ac, leading to chromatin compaction [67]. In recent years, advancements in epigenetic research, driven by high-throughput technologies, have greatly expanded our understanding of the role of these enzymes in oncogenesis [69,70]. Among the various epigenetic modulators, one particularly noteworthy and reversible modification that has garnered significant research interest is m6A RNA modification, especially in the context of cancer development [71]. The m6A modification is not only implicated in cancer pathogenesis but also plays a role in drug response and immune modulation [72,73]. This methylation of adenosine at the N6 position has been identified as one of the most crucial mRNA modifications. Subsequent studies have underscored its importance in numerous biological processes, both under normal and pathological conditions [72,74]. It has been discovered that the function of m6A is regulated by specific RNA methyltransferases, referred to as “writers,” and demethylases, known as “erasers.” A balanced ratio between these regulators is essential for maintaining proper m6A modification levels. The dysregulation of this balance, resulting from mutations or altered expression levels, can contribute to the development of various diseases, including cancer [72]. In ovarian cancer, it has been reported that the m6A-related RNA signature holds potential as a prognostic factor [71]. A similar impact on immune modulation was observed in the study by Gu et al., which showed that a low level of m6A modification was associated with immune system activation and enhanced response to immunotherapy, while a high level of m6A modification correlated with tumor progression. A negative correlation was also found between the stage of OC and m6A modification levels. In OC patients with a low m6A modification score, a high expression of genes involved in immune response modulation was observed, including genes related to human leukocyte antigens, immune activation, and immune checkpoint molecules [75]. Additionally, it has been demonstrated that m6A modification negatively regulates the expression of ERα by influencing RNA demethylases. When RNA demethylases are reduced, m6A levels increase, leading to the enhanced methylation of ERα mRNA and consequently decreased ERα protein translation, as observed in cholangiocarcinoma. Similar findings, suggesting a role in immune response modulation, have been reported in osteosarcoma and breast cancer [76,77]. Although the precise role of m6A modifications in OC is not yet fully understood, existing data suggest that m6A modification regulators, by influencing epigenetic changes, could play a critical role in OC therapy, especially in the context of immunotherapeutics. This may help identify patient subgroups most likely to benefit from such treatments [78].

### 3.1. Epigenetic Mechanisms Involved in Estrogen Signaling in Ovarian Cancer

Estrogen signaling can be regulated by epigenetic mechanisms such as DNA methylation and histone modifications. These epigenetic changes can influence the expression or activity of estrogen receptors (ERα, ERβ, and GPER1), estrogen receptor co-regulators, and downstream effectors like cyclins, which in turn promote the estrogen (E2)-dependent proliferation of OC cells [79].

#### 3.1.1. Epigenetic Regulation of ERα Expression and Activity in Ovarian Cancer

ERα functions as a ligand-inducible transcription factor that mediates various biological processes in response to estrogen stimulation [80]. Upon binding to its ligand, ERα translocates to the nucleus, where it binds to estrogen response elements (EREs)—specific DNA sequences that are often subject to epigenetic modifications or particular histone modifications, which mark them as transcriptional enhancers [81]. After binding to EREs, ERα functions as a co-factor recruitment molecule that targets the transcription of target genes [82]. Promoter hypermethylation, particularly in the context of DNA methyltransferase (DNMT) activity, is known to be associated with a decreased expression of estrogen receptor alpha (ERα) in various cancers. DNA methylation in the promoter region of the ERα gene *ESR1* can silence its expression, which is observed in several cancer types, including ovarian and endometrial cancers [83]. Studies have shown that an overexpression of DNMT1 and DNMT3A, enzymes responsible for methylation, correlates with lower ERα levels and poor prognosis in cancer [84]. *ESR1* silencing has also been observed in other cancers, including endometrial cancer, where *ESR1* promoter methylation led to decreased ERα expression [85]. In vitro, it was reported that *ESR1* demethylation by 5-aza-2′-deoxycytidine (DAC) restored ERα expression in an ERα-negative cancer cell line [86]. Although this suggests that the epigenetic silencing of *ESR1* via DNA methylation is reversible, and demethylation can potentially restore ERα expression, a clinical use, e.g., aiming at re-sensitizing cancers to hormone therapies, is not possible, since demethylating drugs are totally unspecific and thus could lead to an increased expression of pathobiologically active genes like oncogenes.

In ovarian cancer, the dysregulation of ERα via epigenetic modifications is increasingly recognized as a key factor influencing tumor progression, therapy resistance, and prognosis. Epigenetic changes like DNA methylation and histone modifications modulate ERα expression and activity in the tumor microenvironment. The hypermethylation of the *ESR1* promoter is a hallmark of reduced ERα expression in ovarian cancer. However, the *ESR1* methylation status plays different roles in high-grade OC and in low-grade OC. Although the majority of high-grade serous ovarian and endometrioid ovarian cancers express different, IHC-detectable levels of ERα, the expression of this receptor is generally higher in low-grade OC. Both the hypo- and hypermethylation of the *ESR1* promoter were observed in OC. The hypermethylation of the *ESR1* gene, which is also present in many OC cell lines, results in reduced gene expression, but does not necessarily lead to an ERα-negative IHC status, whereas *ESR1* hypomethylation generally increases ERα levels [87]. A study comparing both the methylome and transcriptome of not further characterized OC samples and normal ovarian tissues identified *ESR1* to be among the genes which were the most significantly hypomethylated and overexpressed in OC. *ESR1* hypomethylation leading to high ERα levels was associated with longer overall survival (OS) and post-progression survival (PPS) in OC patients [88]. In another study differentiating high- and low-grade OC, the hypermethylation of *ESR1* was associated with a poorer prognosis in low-grade OC, but not in HGSOC [89]. This observation is thought to result from the fact that low-grade OC generally is more hormone-dependent than HGSOC, being primarily driven by other pathways. *ESR1* hypermethylation resulting in reduced ERα levels is thought to be associated with a poorer prognosis, particularly in LGOC, because it might trigger the usage of compensatory, more aggressive growth and survival pathways, which are already dominant in HGSOC. ERα-negative ovarian cancers often show an increased activation of pathways like PI3K/AKT/mTOR or NFκB signaling, contributing to enhanced proliferation, shorter survival, and resistance to apoptosis. Furthermore, studies indicate that ERα plays a role in maintaining epithelial characteristics of OC cells. Its loss due to *ESR1* hypermethylation can promote epithelial-to-mesenchymal transition (EMT), a process associated with increased invasiveness, migration, and metastatic capacity. The histone methyltransferase DOT1L has been proposed to be a functional component of ERα signaling in OC. ERα and DOT1L were shown to be highly co-expressed in ERα-positive, chemotherapy-resistant OC cells. DOT1L controls chromatin functions involved in tumor initiation and progression and has been proposed as a prognostic OC biomarker. The inhibition of DOT1L or ERα exerted growth inhibitory effects in ERα-positive, hormone-dependent OC [90].

Another level of complexity in ERα epigenetics was added by the identification of the MegaTrans (Mega Transcriptional) complex, which was demonstrated to modulate both ERα activity and expression. The MegaTrans complex epigenetically regulates the transcriptional activity of ERα by influencing chromatin accessibility and enhancer activity at its target genes. In estrogen-responsive cells, the MegaTrans complex amplifies the activity of ERα by enabling the formation of super-enhancers, clusters of highly active enhancers enriched in histone acetylation marks, which drive a robust transcription of estrogen-responsive genes. Additionally, by epigenetic feedback, the MegaTrans complex activates the expression of the *ESR1* gene by histone acetylation at its regulatory regions. The MegaTrans complex is a large transcriptional regulatory assembly consisting of transcription factors, coregulators, and histone modifying enzymes that integrates signals from various pathways to modulate gene expression. It connects extracellular and intracellular signals to trigger epigenetic modifications such as histone acetylation to facilitate dynamic changes in chromatin structure and transcriptional activity. ERα is a critical component of the MegaTrans complex, which connects ERα signaling with other pathways, e.g., NFκB, enabling a synergistic regulation of histone acetylation and transcription. Upon estrogen binding, ERα recruits the MegaTrans complex, which includes histone acetyltransferases like CBP/p300. These enzymes deposit histone acetylation marks, such as H3K27ac, at promoter or enhancer regions, serving as important epigenetic signals for the recruitment of bromodomain-containing proteins, which regulate chromatin compaction and promote transcriptional activation. The MegaTrans complex also coordinates with other transcription factors, enabling the establishment of super-enhancers, regions of densely acetylated chromatin that amplify the transcription of genes critical for cell proliferation and survival [91]. This mechanism is particularly relevant in estrogen-driven cancers, where the dysregulation of ERα signaling and associated histone acetylation marks contribute to oncogenesis and therapy resistance. The MegaTrans complex, which has been shown to play an important role in aberrant ERα-regulated gene expression in breast cancer, also plays a major role in OC [92]. In a study aiming to discover new regulatory factors causing distinct biological properties of HGSOCs and serous borderline tumors (SBTs), several factors were identified which are known to cooperate with and predict the presence of ERα and which are known to form the MegaTrans complex. The results of this study implicated an estrogen-responsive regulatory network in the differential gene expression of HGSOC and SBT and was the first demonstrating the contribution of the MegaTrans complex in distinct biological trajectories of different OC subtypes [93].

#### 3.1.2. Epigenetic Regulation of ERβ Expression and Transcriptional Activity in Ovarian Cancer

In ovarian cancer, like in other cancer entities, the expression of the *ESR2* gene coding for ERβ is reduced compared to normal tissues, and low levels of this receptor have been reported to be associated with a more aggressive cancer phenotype and with poor OC survival due to the role of ERβ as a tumor suppressor [31,40,44,94]. The *ESR2* gene is known to be epigenetically regulated both by promoter methylation and by histone modifications, affecting the transcriptional activity and gene expression of ERβ. However, few studies addressed the epigenetic regulation of ERβ in OC. Early studies on breast cancer tissues demonstrated a high level of *ESR2* promoter methylation, leading to the down-regulation or loss of ERβ expression in invasive breast cancer, but not in the normal mammary gland [95]. Furthermore, *ESR2* activity and expression both are regulated by histone modifications, e.g., triggered by KDM6B histone demethylase [96]. In many in vitro studies, drugs like DNMT inhibitors exerting de-methylating effects or HDAC inhibitors reducing histone acetylation, leading to a more accessible chromatin structure allowing higher transcriptional activity, have been employed to indirectly examine and counteract the epigenetic silencing of ERβ [97,98]. In OC, ERβ activity has been reported to be regulated by the modulation of histone acetylation marks in the promoter of ERβ target genes [99]. In human OC cells, an analysis of the *ESR2* promoter revealed 13 CpG methylation sites. The demethylation of these sites could be triggered by treatment with 5-aza-2′-deoxycytidine (5-aza-dC), an inhibitor of DNA and RNA methyltransferases. In this study, it was shown that the treatment of OC cells by epigenetic agents—5-AzaC and histone deacetylase inhibitor trichostatin A—leads to the reactivation of ERβ expression and activity and thereby triggers the inhibition of ovarian cell proliferation. This study demonstrated that the down-regulation of ERβ expression and activity in OC cells results both from promoter hypermethylation and histone acetylation [100]. A deeper analysis of *ESR2* methylation examining both promoter regions of the *ESR2* gene, which are termed 0K and 0N, showed that promoter 0N was rarely methylated in normal ovarian tissues, but extensively methylated in OC cell lines and tissues, whereas the promoter 0K was unmethylated in both normal and malignant ovarian cells and tissues. The same study also examined the expression of the ERβ splice variants, demonstrating that the hypermethylation of the 0N promoter was associated with a loss of the expression of the variants ERβ1, ERβ2, and ERβ4 in ovarian carcinoma cells and tissues. Treatment with the demethylating agent 5-aza-dC restored the expression of these ERβ splice variants. However, ERβ5 expression was not decreased in clear cell adenocarcinoma when compared to the normal ovary and was not found to be associated with the methylation status [101]. The data from this study clearly support the functional connection of promoter hypermethylation with a loss of ERβ expression, which is observed not only in OC, but also in other cancer entities like breast and prostate cancer [102,103].

#### 3.1.3. Epigenetic Regulation of GPER1 Expression and Activity in Ovarian Cancer

The G protein-coupled estrogen receptor (GPER1) was found to be expressed in the normal ovary and in OC and its function has crucial implications in both physiological and pathological processes [104,105]. GPER1 expression was reported to be lower in OC tissue than in benign and low-malignancy ovarian tumors. However, as discussed above, conflicting data exist about the correlation of GPER1 expression in OC patients with survival [48,49,50]. A loss or reduction of GPER1 expression was reported to occur in 20–50% of OC cases [49,106]. A recent study using data from the cancer genome atlas (TCGA) and the genotype-tissue expression (GTEx) databases for analysing the expression profile of estrogen receptors through gene expression profiling interactive analysis (GEPIA) found GPER1 downregulation in OC, which, however, was not statistically significant. In contrast, in the majority of cancer entities including breast and endometrial cancer, GPER1 expression was significantly reduced and associated with the hypermethylation of the *GPER1* gene promoter [107]. Other studies confirmed *GPER1* expression to be reduced by hypermethylation in various cancers, like breast cancer [108,109]. In ovarian cancer, a recent study suggests that *GPER1* expression is epigenetically activated by histone H3 trimethylation (H3K4me) (Table 1). This histone H3 modification is one of the most recognized epigenetic marks of active transcription [110]. Although the role of histone marks in various cell types has been extensively explored, it is still not fully understood in cancer. However, H3K4me3 marks are an epigenetic signal, which in normal cells is associated with increased transcription elongation and enhancer activity at tumor-suppressor genes like TP53 and PTEN. In cancer cells, these H3K4me3 marks and enhancer activity are reduced, leading to the decreased transcription of tumor suppressor genes and elevated cancer cell proliferation [110,111,112]. In ovarian cancer, however, high levels of H3K4me3 marks were shown to be associated with high *GPER1* expression, leading to a better prognosis of GPER1-positive OC patients. Given that GPER1 was suggested to act as tumor suppressor in the ovary, this observation might be explained by the known activation of tumor suppressor genes by H3K4me3 marks [113].

#### 3.1.4. Epigenetic Regulation of Estrogen Receptor Target Genes in Ovarian Cancer

In particular, the estrogen-driven activation of the nuclear receptors ERα and ERβ, which are ligand-inducible transcription factors, induces the transcription of their target genes, which are mediators of estrogen signaling. In studies on breast cancer, but also OC cells, ERα itself was recognized as a component of the epigenetic acting MegaTrans complex, which contains histone acetyltransferases (HATs), and deposits histone acetylation marks, such as H3K27ac, at promoter or enhancer regions, and as such regulates chromatin compaction and activates transcription, particularly of estrogen-dependent genes. This epigenetic mechanism was reported to activate the transcription of estrogen target genes like *TFF1* (PS2), *FOXA1* and *GREB1* [91]. Cyclin D1 coded by the gene *CCND1* is an important target gene of ERα and mediator of estrogen-induced cellular proliferation. Recently, it has been shown that *CCND1* is regulated by epigenetic mechanisms. First, *CCND1* expression is epigenetically activated by protein arginine methyltransferase 6 (PRMT6), which generates histone modification mark H3R2me2a, in conjunction with transcription factor LEF [114]. In ovarian cancer, the *CCND1* promoter was reported to be hypomethylated, leading to enhanced gene expression and poor prognosis [115]. The *CDH1* gene coding for E-cadherin was described as an estrogen target gene being downregulated by ERα in OC [116]. This gene was found to be hypermethylated and downregulated in OC patients, leading to enhanced metastasis [117,118]. While *PTEN* is not a direct target of ERα, estrogen signaling can affect its expression indirectly. Estrogen can downregulate *PTEN* via pathways that activate AKT or other signaling cascades promoting cell survival and proliferation. In certain cancers, reduced PTEN levels due to estrogen action are linked to enhanced tumorigenesis. Epigenetic mechanisms such as promoter methylation also modulate *PTEN* expression in cancers, including OC [119]. Antiapoptotic factor Bcl-2 is a direct target of ERα. Its expression is upregulated by estrogen, contributing to cell survival in hormone-sensitive cancers. In ovarian cancer, the expression of Bcl-2 was demonstrated to be epigenetically suppressed by HDAC1, which deacetylates histone H3K9,14 marks across Bcl-2 regulatory regions, resulting in reduced Bcl-2 transcription [120]. FOXA1, a key determinant of estrogen receptor function, which enables ERα interactions with chromatin, was reported to induce epithelial OC tumorigenesis and progression [121]. Recently, *FOXA1* was reported to be epigenetically modulated by histone deacetylase HDAC3 in the progression of epithelial ovarian carcinoma [122].

**Table 1:** Modulation of estrogen signaling by DNA methylation or histone modification in OC.

Epigenetic Mechanism	Gene	References
Promoter methylation	*ESR1*	[86,87,88,89]
	*ESR2*	[99,100]
	*GPER1*	[59]
	*CDH1*	[117,118]
	*CCND1*	[114]
	*PTEN*	[119]
Histone modification	*ESR1*	[89,91,92]
	*ESR2*	[98,99]
	*GPER1*	[109,112]
	*TFF1*	[90]
	*FOXA1*	[90,122]
	*GREB1*	[90]
	*CCND1*	[113]
	*BCL2*	[120]

## 4. Drugs with Epigenetic Functions: Are They Promising Tools for OC Therapy?

The epigenetic changes occurring during the process of ovarian carcinogenesis, including those altering estrogen signaling, are increasingly understood and known. Given that epigenetic mechanisms play an important role in OC development and progression, e.g., activating the expression of oncogenes or reducing the activity of tumor-suppressor genes, they seem to be promising targets for OC therapy. The anti-tumoral effect of drugs with epigenetic functions has been thoroughly examined in in vitro studies employing cell lines of various cancer entities, including OC, or studies using mouse models, and has often led to impressive results [123,124,125,126,127,128,129,130,131,132]. However, the major problem regarding the clinical use of “epidrugs” is their totally unspecific action. They can change the expression of every gene in all tissues of the human body. They can even have the potential to activate oncogenes or to suppress tumor suppressor genes. This lack of specificity of “epidrugs” can thus cause major side effects leading to a high toxicity or to the development of diseases, which might come to light months or years after the treatment with such drugs, since the pathogenesis of many diseases takes time. However, initial clinical studies have examined the effect of “epidrugs” like HDAC or DNMT inhibitors, mostly in combination with standard treatments, on the survival of patients with various cancer types [133,134]. In women with recurrent OCs, several trials using HDAC or DNMT inhibitors in combination with standard therapy regimens have been performed to date, mostly reporting severe side effects (reviewed in [135]). Other studies showed more promising results. A phase I-II trial investigated the DNMT inhibitor decitabine and carboplatin in women with platinum-resistant OC. To minimize toxicity and enhance the demethylating properties of decitabine, the regimen included low daily doses of decitabine for five days prior to carboplatin. The combination was found to be tolerable and clinically promising [136,137]. With regard to HDAC inhibitors, Vorinostat was tested as a single agent in patients with recurrent OC relapsing within 12 months after platinum-based therapy in a Gynecologic Oncology Group (GOG) trial [138]. Out of twenty-seven women enrolled, two were free of progression at 6 months, deeming the drug insufficiently active as a single agent. Additionally, significant hematological toxicity was observed. In another trial, Belinostat had modest clinical activity, but high toxicity, among 32 patients with recurrent OC. One partial response and 10 patients with stable disease were reported, with increased activity being noted in patients with low-grade serous OC [139].

In conclusion, clinical studies employing “epidrugs” like inhibitors of HDACs or DNTMs, often showed severe toxicity, as expected considering the lack of the specificity of these drugs. Thus, prolonged studies on animal models are needed before the use of such substances can be considered to be ethically justifiable for the treatment of cancer patients. A promising strategy to make “epidrugs” more specific could be the modification of their structure limiting their action to the target gene by the addition of a short but specific DNA sequence which can guide the drug/DNA complex to the target site. However, novel insights into the epigenetic mechanisms involved in the development and progression of various cancer entities have the potential to open new avenues for novel treatment strategies for OC.
